# End-of-Life Preferences: A Randomized Trial of Framing Comfort Care as Refusal of Treatment in the Context of COVID-19

**DOI:** 10.1177/0272989X231171139

**Published:** 2023-05-18

**Authors:** Juliet S. Hodges, Lilia V. Stoyanova, Matteo M. Galizzi

**Affiliations:** Department of Psychological and Behavioural Science, London School of Economics, London, UK; Department of Psychological and Behavioural Science, London School of Economics, London, UK; Department of Psychological and Behavioural Science, London School of Economics, London, UK

**Keywords:** advance directive, COVID-19, nudge, behavioral economics, defaults, framing, priming

## Abstract

**Background:**

Rates of advance directive (AD) completion in the United Kingdom are lower than in the United States and other western European countries, which is especially concerning in light of the COVID-19 pandemic. UK residents typically complete an advance decision to refuse care (ADRT), whereas US versions of ADs present a more neutral choice between comfort-oriented or life-prolonging care. The purpose of this study is to test whether this framing affects decision making for end-of-life care and if this is affected by exposure to information about the COVID-19 pandemic.

**Methods:**

In an online experiment, 801 UK-based respondents were randomly allocated to document their preferences for end-of-life care in a 2 (US AD or UK ADRT) by 2 (presence or absence of COVID-19 prime) between-subjects factorial design.

**Results:**

Most (74.8%) of participants across all conditions chose comfort-oriented care. However, framing comfort care as a refusal of treatment made respondents significantly less likely to choose it (65.4% v. 84.1%, *P* < 0.001). This effect was exacerbated by priming participants to think about COVID-19: those completing an ADRT were significantly more likely to choose life-prolonging care when exposed to the COVID-19 prime (39.8% v. 29.6%, *P* = 0.032). Subgroup analyses revealed these effects differed by age, with older participants’ choices influenced more by COVID-19 while younger participants were more affected by the AD framing.

**Conclusions:**

The UK ADRT significantly reduced the proportion of participants choosing comfort-oriented care, an effect that was heightened in the presence of information about COVID-19. This suggests the current way end-of-life care wishes are documented in the United Kingdom could affect people’s choices in a way that does not align with their preferences, especially in the context of the COVID-19 pandemic.

**Highlights:**

- Participants completing an AD framed as an advance decision to refuse treatment were significantly less likely to choose comfort-oriented care than participants completing an AD with a neutral choice between comfort-oriented and life-prolonging care.
- Exposure to a COVID-19 prime had an interactive effect on documented preferences in the refusal of treatment condition, with these participants even less likely to choose comfort-oriented care.
- Policy makers and organizations that design templates for advance care planning, particularly in the time of the COVID-19 pandemic, should be aware how the framing of these forms can influence decisions.

Evidence suggests that people who have documented their wishes for end-of-life care experience a better quality of death. Specifically, people who have completed advance decisions (ADs) are more likely to receive their preferred care and less likely to die in hospital, and there are fewer communication issues with their surrogate decision makers.^1^ However, the percentage of the UK population who already have an AD is very low. A poll found that only 4% of people in England and 2% in Wales have completed an AD,^2^ consistent with a study in one hospital that found only 4% of 9,000 patients who died there had an advance care plan.^3^ Other European countries have completion rates of up to 20%, such as Germany, where the uptake is about 10%,^2^ while more than one-third of the US population are estimated to have one.^4^

While there are cultural and contextual differences between the United States and the United Kingdom that could affect decisions around end-of-life care,^5–7^ insights from behavioral science suggest the disparity could be driven also by the typical wording of the AD form. In the United States, patients completing an AD were found to be influenced by whether the form had a default toward comfort-oriented or life-extending care.^8^ This was even true in a later study in which participants were given the opportunity to change their minds after being explicitly made aware of the default and how it may have affected their decision.^9^

While the AD presents a neutral choice between comfort and life-prolonging care, UK citizens are given the option to complete an advance decision to refuse treatment (ADRT). In other words, comfort care is framed as having no treatment at all. It seems likely this framing could have an impact on how people perceive it: studies have shown that patients will accept a treatment that has no chance of improving their condition when the other option is watchful waiting—particularly when the latter is described as “doing nothing.”^10^ Furthermore, UK citizens would complete the form only if they wanted comfort care; there is no option to select care to prolong life. This implies the default in the United Kingdom is for physicians to provide life-prolonging care and people tend to go along with the default option. Therefore, the low rates of ADRT completion in the United Kingdom could be a result of the negative framing of comfort care and implicit default of life-prolonging care.

These decisions have not only become increasingly relevant in light of the COVID-19 pandemic but could also be influenced by exposure to news about infections, hospitalizations, and deaths. Increased mortality salience following deadly disasters has been linked to an increase in risk-seeking behavior,^11,12^ which could influence how people perceive the choice between comfort and life-prolonging care. Accepting life-prolonging treatments could be seen as the riskier option, as people are risking more pain and suffering for the chance of a longer life. Comfort care involves less uncertainty but also the likely outcome of a shorter life. There is also some evidence that the threat of infectious disease increases the tendency to conform, a “behavioral immune system” response that may have evolved to keep outsiders and communicable diseases away.^13–15^ An increase in conformity could lead more people to choose life-prolonging care, as it is implicitly the default option under the UK’s ADRT system.

It seems unlikely that all but 4% of the UK population would prefer life-prolonging care, given statistics from previous studies and culturally similar countries. For example, one study found that 65% of respondents in England would choose improving the quality of their life over extending it as the priority for their treatment if they were diagnosed with a serious illness.^16^ This has important implications: this simple framing could be responsible for people in the United Kingdom being less likely to engage with end-of-life planning in general and less likely to have a death consistent with their preferences, particularly if they would like to prioritize comfort but not to refuse treatment. The influence of this frame on people’s responses to questions about end-of-life care has not, to our knowledge, been tested experimentally before.

The aim of this study is, first, to measure the effect of different form templates on decisions about end-of-life care, comparing the US AD with the UK ADRT. In the United States, standard AD forms give a free choice between prioritizing comfort or life-prolonging care. The ADRT form commonly used in the United Kingdom, however, only gives the option of comfort care for anyone completing the form. This study is the first of its kind to experimentally examine the effect of positioning comfort care as refusing treatment in the UK frame, relative to presenting the choices more neutrally in the US frame.

The second aim of the study is to identify what, if any, effect priming participants to think about the COVID-19 pandemic has on these choices. The data collection was undertaken in September 2020, between the United Kingdom’s peaks of COVID-19 cases and before the first vaccines were distributed, meaning the threat of the virus was still very salient and real. Therefore, at the beginning of each form template, we add a second manipulation to include (or not) a prime with information about COVID-19.

We hypothesized that most participants in all conditions would choose comfort care over life-prolonging care in line with previous research (hypothesis 1). However, we also hypothesized that participants in the UK condition would be more likely to choose life-prolonging care than participants in the US condition, due to the negative framing of comfort care as refusal of treatment (hypothesis 2). As a result of the increase in mortality salience from reflecting on the pandemic, we predicted that participants exposed to a COVID-19 prime before completing the form would be more likely to choose life-prolonging care than comfort care (hypothesis 3). Finally, we hypothesized there would be an interaction between framing and priming, with participants in the UK frame and COVID-19 prime condition even more likely to choose life-prolonging care (hypothesis 4). This is because we predicted that the UK framing of life-prolonging care as accepting treatment would be perceived as the default option, while the threat of infectious disease from the COVID-19 prime would lead to increased conformity to that norm.

## Methods

A preregistered online randomized experiment was conducted in which participants documented their preferences for end-of-life care, in the event of being unable to communicate their wishes (https://osf.io/cqk42). The impact of framing and COVID-19 prime was tested using a 2 (UK-style ARDT v. US-style AD) by 2 (presence v. absence of COVID-19 information) factorial between-subjects design.

### Procedure and Conditions

Data were collected in September 2020. Participants were recruited through Prolific Academic and paid 88p for their participation (on average, £9.60 per hour). The survey itself was hosted on Qualtrics, where participants were randomly allocated to 1 of the 4 conditions in equally sized groups. At the start of the experiment, participants in the COVID-19 prime condition read a short summary of symptoms, complications and death rates of the virus, while participants in the no prime condition read a generic introduction to advance care planning. When completing the AD, participants in the UK condition were given a choice between accepting or refusing treatment, while participants in the US condition chose between comfort care or life-prolonging care.

All participants completed 10 questions on their version of the AD. First, they chose their preference for the overall goal of their care (question 1—see below for instructions and options). Next, they chose their preferences in case of being diagnosed with the following 4 illnesses: dementia, a brain injury, a disease of the central nervous system (CNS), and other terminal illnesses (questions 2–5). Third, they chose their preference for accepting or rejecting the following 5 treatments: cardiopulmonary resuscitation (CPR), admission to the intensive care unit (ICU), mechanical ventilation, kidney dialysis, and feeding tube insertion (questions 6–10). These questions were presented in the same order for every participant.

For example, the instructions and options for the overall preference for their care are as follows:

*Question 1. If I have a condition where I have no reasonable expectation of recovery or chance of regaining a meaningful quality of life, my instructions for the overall goal of my care are as follows*:

#### UK condition

- *I would like to exercise my right to refuse treatment. I want my health care providers and agent to pursue treatments that help relieve my pain and suffering, even if that means I might not live as long.*
- *I do not want to refuse treatment and would like to accept the care available to me. I want my health care providers and agent to pursue treatments to prolong my life, even if that means I might have more pain or suffering.*

#### US condition

- *I want my health care providers and agent to pursue treatments that help relieve my pain and suffering, even if that means I might not live as long.*
- *I want my health care providers and agent to pursue treatments to prolong my life, even if that means I might have more pain or suffering.*

Following completion of the form, participants were asked additional questions about attitudes toward and experiences of health care, their concerns and experiences with COVID-19, and demographic information such as age, sex, education, and ethnicity.

### Outcomes

The primary outcome is whether participants choose comfort care or life-prolonging care, in 3 domains: 1) as the overall preference for their care, 2) in the case of 4 specific illnesses (being diagnosed with dementia, a brain injury, a disease of the CNS, and other terminal illnesses), and 3) in the case of 5 specific treatments (CPR, admission to the ICU, mechanical ventilation, kidney dialysis, and feeding tube insertion). The 4 conditions are based on the Compassion in Dying living will template,^17^ while the 5 treatments are based on previous studies.^9^

There are also several secondary outcomes and control variables. First, a behavioral measure recorded whether participants clicked on a link to the AD page on the NHS website after completing the form. Second, questions were asked about relevant experiences and attitudes, such as their current health, access to private health insurance, being admitted to an ICU, the death of a loved one, and preferences for decision making with a doctor. Third, questions about coronavirus itself were included: concerns about contracting it and getting seriously ill, worries about loved ones getting seriously ill, whether the participant or any of their loved ones had had it, and how severe those cases were. Finally, demographic information was collected, including age, gender, ethnicity, religion, and education, as it is possible these factors could also influence end-of-life decisions.

### Power Calculation

The necessary sample size to detect a minimum effect size of 0.3 (a small to medium minimum effect size), with 0.80 power and a standard 0.05 alpha error probability, was calculated using G*Power for a Wilcoxon-Mann-Whitney nonparametric test and a logistic regression. The sample size per group was between 160 and 184, so, to be conservative, the study aimed to recruit 200 participants per group, with 800 in total.

### Statistical Analyses

Data were analysed using Wilcoxon-Mann-Whitney nonparametric tests for differences-in-proportions. Further analysis was performed using logistic regressions to determine the main effects of country frame and prime, their interactive effects, and control for additional variables such as age, gender, and ethnicity. To account for multiple hypotheses testing, a Bonferroni-corrected significance level of 0.005 was used for all analyses. Analysis was conducted using Stata 17.0.

## Results

### Participants

A total of 801 UK resident participants were recruited through Prolific Academic, of whom 60.6% were female, with an average age of 34.08 ± 13.1 y (ranging from 18 to 80 y). Ethnicity was predominantly White at 84.1%, 8.5% Asian, 2.5% Black, 3.9% mixed race, and 1% other. Table 1 shows the sociodemographic characteristics by experimental condition.

**Table 1:** Participant Characteristics by Experimental Condition

	United States		United Kingdom		
	COVID-19	No COVID-19	COVID-19	No COVID-19	Total
*n*	206	196	196	203	801
Overall choice					
Comfort, *n* (%)	177 (85.9)	161 (82.1)	118 (60.2)	143 (70.4)	599 (74.8)
Prolong, *n* (%)	29 (14.1)	35 (17.9)	78 (39.8)	60 (29.6)	202 (25.2)
Mean (*s*) age, y	33.81 (12.9)	33.68 (14.0)	34.24 (13.2)	34.59 (12.4)	34.08 (13.1)
Female, *n* (%)	126 (61.2)	132 (67.3)	111 (56.6)	116 (57.1)	485 (60.6)
Ethnicity, *n* (%)					
Asian	10 (4.9)	16 (8.2)	21 (10.7)	21 (10.3)	68 (8.5)
Black	7 (3.4)	3 (1.5)	6 (3.1)	4 (2.0)	20 (2.5)
Mixed	7 (3.4)	5 (2.6)	11 (5.6)	8 (3.9)	31 (3.9)
White	178 (86.4)	170 (86.7)	158 (80.6)	168 (82.8)	674 (84.1)
Other	4 (1.9)	2 (1.0)	0 (0)	2 (1.0)	8 (1.0)
Religion, *n* (%)					
Buddhist	3 (1.5)	2 (1.0)	1 (0.5)	1 (0.5)	7 (0.9)
Christian	60 (29.1)	52 (26.5)	49 (25.0)	65 (32.0)	226 (28.2)
Hindu	0 (0.0)	7 (3.6)	2 (1.0)	3 (1.48)	12 (1.5)
Jewish	0 (0.0)	1 (0.5)	0 (0.0)	0 (0.0)	1 (0.1)
Muslim	5 (2.4)	2 (1.0)	11 (5.6)	6 (3.0)	24 (3.0)
Sikh	3 (1.5)	1 (0.5)	1 (0.5)	1 (0.5)	6 (0.8)
Other	4 (1.9)	8 (4.1)	3 (1.5)	4 (2.0)	19 (2.4)
None	131 (63.6)	123 (62.8)	129 (65.8)	123 (60.6)	506 (63.2)
Education, *n* (%)					
Secondary	16 (7.8)	19 (9.7)	24 (12.2)	24 (11.8)	83 (10.4)
A-levels	44 (21.4)	47 (24.0)	46 (23.5)	47 (23.2)	184 (23.0)
Undergraduate	79 (38.4)	77 (39.3)	67 (34.2)	69 (34.0)	292 (36.5)
Postgraduate	24 (11.7)	25 (12.8)	27 (13.8)	31 (15.3)	107 (13.4)
Doctoral	8 (3.9)	1 (0.5)	5 (2.6)	3 (1.5)	17 (2.1)
Vocational	15 (7.3)	16 (8.2)	17 (8.7)	18 (8.9)	66 (8.2)
Professional	17 (8.3)	9 (4.6)	7 (3.6)	8 (3.9)	41 (5.1)
Other	1 (0.5)	1 (0.5)	3 (1.5)	2 (1.0)	7 (0.9)
No formal qual	2 (1.0)	1 (0.5)	0 (0.0)	1 (0.5)	4 (0.5)

### Effects of Country Frame and Coronavirus Prime on Question 1—Overall Goal of Care

On average, 74.8% of participants selected comfort for the overall goal of their care, which supports our first hypothesis. A significant main effect of the country frame was observed (supporting hypothesis 2): 84.1% of participants in the US AD condition chose comfort care, while 65.4% of participants in the UK ADRT condition chose comfort (z = −6.08, *P* < 0.001).

There was no significant effect of the COVID-19 prime overall (not supporting hypothesis 3): 73.4% of participants who were primed chose comfort, compared with 76.2% of participants who did not receive a prime (z = −0.91, *P* = 0.36).

**Figure 1:**
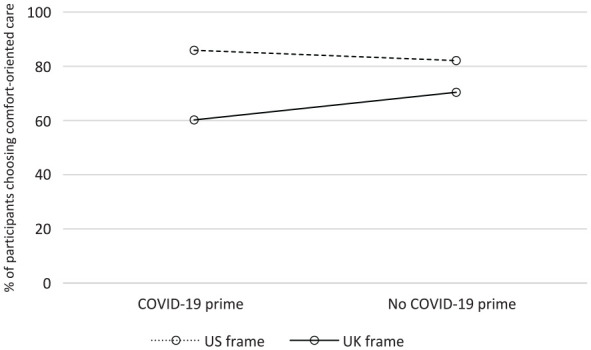
Percentage of participants choosing comfort care for question 1 (overall goal of care) in the US condition and the UK condition.

There was a significant interaction between the country frame and COVID-19 prime, supporting hypothesis 4 (see Figure 1). In the US AD condition, the prime did not significantly alter participants’ choices: 85.9% chose comfort when primed, compared with 82.1% without a prime (z = 1.03, *P* = 0.30). In the UK ADRT condition, the COVID-19 prime made participants more likely to choose life-prolonging care: 60.2% chose comfort care when primed, compared with 70.4% without a prime, although this effect was not significant after Bonferroni correction (z = −2.15, *P* = 0.032).

This relationship was confirmed in a series of logistic regressions modeling the probability of choosing life-prolonging care (see Table 2). These regressions were performed to test the effect of each condition separately, in combination, and with exogenous individual characteristics. Estimated average marginal effects showed participants in the UK condition were 18.5% more likely to choose life-prolonging care (95% z = 6.39, *P* < 0.001). Estimated average marginal effects showed participants in the United Kingdom and COVID-19 condition were 13.2% more likely to choose life-prolonging care (95% z = 2.15, *P* = 0.032), although again this was not significant after Bonferroni correction for multiple hypotheses testing.

**Table 2:** Logistic regression of country frame, COVID-19 prime, and additional demographic and attitudinal variables for prolonging life as the overall goal of care^a^

Condition and Participant Characteristics	Prolonging Life as Overall Goal of Care, Coefficient (SE)				
	Model 1	Model 2	Model 3	Model 4	Model 5
UK country frame	1.03 (0.17)***		1.03 (0.17)***	0.66 (0.24)**	0.71 (0.25)**
COVID-19 prime		0.15 (0.16)	0.18 (0.17)	−0.28 (0.27)	−0.28 (0.28)
UK*COVID-19 prime				0.74 (0.35)*	0.72 (0.36)*
Age (>40 y)					−0.31 (0.08)***
Gender					0.27 (0.16)
Ethnicity					−0.13 (0.09)
Religious importance					−0.41 (0.14)**
Concerns about COVID-19					−0.27 (0.09)**
Constant	−1.66 (0.14)***	−1.16 (0.12)***	−1.76 (0.16)***	−1.53 (0.19)***	1.07 (0.54)
Observations	801	801	801	801	801
*R* ^2^	0.04	0.00	0.04	0.05	0.09

SE, standard error.

a Model 1 shows the impact of only the country frame condition (US v. UK). Model 2 is based only on the COVID-19 prime condition (presence or absence). Model 3 combines the 2 main effects of country frame and COVID-19 prime. Model 4 combines the 2 main effects and controls for the interaction between them. Model 5 adds exogenous individual characteristics.

* *P* < 0.05; **P < 0.01; ***P < 0.001.

These main results were robust to the introduction in the logistic regressions of a range of controls for individual characteristics (age, gender, ethnicity) and beliefs (e.g., religious importance, concerns about COVID-19). However, when these controls were introduced, the estimated average marginal effects of the UK condition reduced slightly, although they remained significant: participants were 12.0% more likely to choose life-prolonging care compared with 18.5% without controls (z = 2.89, *P* = 0.004).

### Exploratory Analysis for Overall Goal of Care

The logistic regressions with control variables also highlighted further findings for which explicit hypotheses were not included in the preregistration. In particular, older respondents, respondents for whom religion was important, or respondents who were very worried about COVID-19 were more likely to choose comfort for their care. There were no significant effects of gender or ethnicity on end-of-life preferences.

Given the strong influence of age on patient preferences, a subgroup analysis was performed, separating participants above and below the median age and repeating the logistic regression models (see Supplementary Material). This revealed that the overall significance of the control variables was driven by very different influences on the 2 groups. For participants over the median age, age, concerns about COVID-19, and the interaction between the country frame and COVID-19 prime remained significant. However, the importance of religion was not significant for this group, nor was the main effect of the country frame at *P* = 0.07. For the youngest half of participants, the opposite pattern was observed: the effect of country frame remained significant, as did the importance of religion. Interestingly, gender also emerged as a significant influence on end-of-life preferences for this group, with males more likely to indicate they would choose life-prolonging care.

### Effects of Country Frame and COVID-19 Prime on Care in the Case of Specific Illnesses (Questions 2–5)

**Figure 2:**
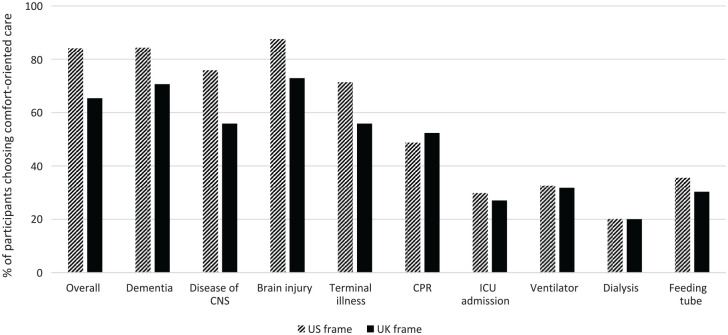
Percentage of participants choosing comfort care in the US and UK frame conditions for their overall goal of care, each specific illness, and each specific treatment.

Further analysis showed that the main effect of country frame was maintained for 3 of the 4 specific illnesses (dementia, CNS, and terminal illness), with participants in the UK condition significantly more likely to choose life-prolonging care (see Figure 2). For dementia, 84.3% of US participants chose comfort care, compared with only 70.7% in the UK frame (z = −4.63, *P* < 0.001). This pattern was also evident for diseases of the CNS (75.9% US v. 55.9% UK, z = −5.96, *P* < 0.001) and terminal illness (71.4% US v. 55.9% UK, z = −4.56, *P* < 0.001).

The estimated marginal effects of the UK country frame on likelihood to choose life-prolonging care ranged from 13.6% for dementia (95% z = 3.28, *P* = 0.001) to 18.0% for terminal illness (95% z = 3.88, *P* < 0.001) and 18.8% for CNS (95% z = 4.24, *P* < 0.001). For brain injury, while participants in the UK condition were also more likely to choose life-prolonging care (87.6% US v. 72.9% UK, z = −5.12, *P* < 0.001), this effect was not robust to correction for multiple hypotheses testing in the logistic regression.

There was no main effect of the COVID-19 prime nor an interaction with the UK frame for the specific illnesses. This was confirmed in further logistic regressions (see Table 3), where the effect of the UK frame remained significant, but there was no effect of COVID-19 prime nor an interaction between them.

**Table 3:** Logistic Regression of Country Frame, COVID-19 Prime and Their Interaction on Preference for Prolonging Life in the Case of Specific Illnesses

	Prolonging Life as Goal of Care for Specific Illnesses, Coefficient (SE)			
	Dementia	Brain Injury	Diseases of CNS	Terminal Illness
UK country frame	0.80 (0.24)**	0.72 (0.26)**	0.88 (0.24)***	0.80 (0.21)***
COVID-19 prime	−0.02 (0.27)	−0.24 (0.30)	−0.20 (0.23)	−0.10 (0.22)
UK*COVID-19 prime	−0.00 (0.35)	0.49 (0.38)	0.06 (0.31)	−0.24 (0.30)
Constant	−1.67 (0.20)***	−1.83 (0.21)***	−1.04 (0.16)***	−0.97 (0.16)***
Observations	801	801	801	801
*R* ^2^	0.03	0.04	0.04	0.02

CNS, central nervous system; SE, standard error.

** *P* < 0.01; ***P < 0.001.

### Effects of Country Frame and Coronavirus Prime on the Use of Specific Medical Treatments (Questions 6–10)

For the 5 specific medical treatments, there was no significant effect of either the country frame or the COVID-19 prime (see Table 4). However, participants across all conditions showed a reversal in their preferences, with most accepting each medical treatment—in other words, choosing life-prolonging care (see Figure 2). This was the lowest for CPR, with 49.4% of all participants choosing to accept that treatment (51.2% US v. 47.6% UK, z = 1.03, *P* = 0.31), while 71.5% accepted admission to ICU (70.2% US v. 72.9% UK, z = −0.87, *P* = 0.38), 67.8% accepted a ventilator (67.4% US v. 68.2% UK, z = −0.23, *P* = 0.82), 79.9% accepted dialysis (79.9% US v. 80.0% UK, z = −0.04, *P* = 0.97), and 67.0% accepted a feeding tube (64.4% US v. 70.0% UK, z = −1.6, *P* = 0.11).

**Table 4:** Logistic Regression of Country Frame, COVID-19 Prime, and Their Interaction on Preference for Prolonging Life in the Case of Specific Medical Treatments

	Prolonging Life as Goal of Care for Specific Medical Treatments, Coefficient (SE)				
	CPR	ICU Admission	Ventilator	Dialysis	Feeding Tube
UK country frame	−0.25 (0.20)	0.20 (0.22)	0.14 (0.21)	0.20 (0.25)	0.21 (0.21)
COVID-19 prime	0.06 (0.20)	0.07 (0.22)	0.19 (0.21)	0.16 (0.25)	0.10 (0.21)
UK*COVID-19 prime	0.21 (0.28)	−0.12 (0.31)	−0.22 (0.30)	−0.39 (0.35)	0.06 (0.30)
Constant	0.02 (0.14)	0.82 (0.15)***	0.63 (0.15)***	1.30 (0.17)***	0.54 (0.15)***
Observations	801	801	801	801	801
*R* ^2^	0.00	0.00	0.00	0.00	0.00

ICU, intensive care unit; SE, standard error.

*** *P* < 0.001.

### Correlations between Choices

Decisions for the overall goal of care, specific illnesses, and specific medical treatments were all highly correlated. Between specific illnesses, these ranged from *r* = 0.55 to 0.73 (*P* = 0.001), while specific medical treatments ranged between 0.39 and 0.85 (*P* < 0.001). With a composite score for all illness questions, there was a strong positive correlation between overall goal and specific illnesses (*r* = 0.56, *P* < 0.001). There was a smaller but still significant correlation between overall goal and a composite score for all medical treatment questions (*r* = 0.36, *P* < 0.001). There was also a moderate correlation between the composite illness and treatment scores (*r* = 0.43, *P* < 0.001).

### Documenting End-of-Life Decisions

The self-reported likelihood of formally documenting end-of-life preferences in the near future did not significantly differ across conditions. In the US condition, 35.8% of participants stated they were likely or very likely to do so, compared with 36.6% in the UK condition (z = 1.33, *P* = 0.18). With a COVID-19 prime, 35.3% of participants were likely or very likely, compared with 37.1% without a prime (z = −0.51, *P* = 0.61). No significant interaction between UK and COVID-19 prime was observed.

For the behavioral measure, only 24 participants (just under 3%) clicked on the link for more information about Advance Decisions. This was lowest in the UK*COVID-19 prime condition, in which only 1.5% of participants clicked on the link. In the other conditions, this number ranged from 3.4 to 3.6%. Again, there was no main effect of country (z = 0.81, *P* = 0.42) or of COVID-19 prime (z = 0.85, *P* = 0.40), nor an interaction between the two, on whether or not participants clicked on the link.

## Discussion

This study is the first of its kind to experimentally examine the difference between a US-style AD, in which the choice between comfort and life-prolonging care is presented neutrally, and the ADRT used in the United Kingdom, where comfort care is framed as refusing treatment. This was combined with a COVID-19 prime, to reflect the effect the global pandemic might have had on end-of-life care choices. At the time the data were collected in September 2020, COVID-19 cases in the United Kingdom were rising, new restrictions were being announced, and the vaccination program was still months away. As a result, the risk of catching the virus and becoming seriously ill from it would have been very salient when participants were completing this task.

In all conditions, most of our UK-based participants selected comfort care for their overall preference for care and also in the case of their preferences for specific diagnoses. However, this was significantly influenced by the framing of the form participants filled out. As hypothesized, the UK ADRT frame had a significant effect on participants’ choices: framing comfort as refusing treatment significantly reduced the number of participants choosing comfort care, compared with participants who were given a more neutral choice under the US frame. Interestingly, the COVID-19 prime affected only participants given the UK-style form and only for the first question about the overall preferences for their care: respondents in the UK condition and primed with COVID-19 were more likely to choose life-prolonging care. There was no effect on participants in the US condition. This suggests that the effect is not due to a general mortality salience, as this would have had a similar effect in the US frame. Instead, it could be due to an increased desire to conform to standard care as part of the UK manipulation, as the UK frame explicitly stated that standard care focuses on prolonging life. Interestingly, this interaction effect was not observed for the other 4 specific illnesses. One possible explanation to reconcile these findings may be related to the behavioral immune system theory^14^: as the 4 illnesses were all related to noncommunicable diseases, they would not prime conformity as an evolutionary response to slow the spread of infection. Another explanation is that it could simply be due to the fact the COVID-19 prime was shown only once at the beginning of this experiment, so its relatively small impact could have diminished further after that first question.

For specific treatments, the effect of the country frame also disappeared. Furthermore, while most participants preferred comfort overall, when faced with the types of treatments that would usually be considered aggressive for the end of life, the majority chose to accept them. As no additional information was provided about these treatments, it might be due to a lack of knowledge about what they entail or the likely outcome. This is very likely, given the nonclinical sample, and reports of similar misconceptions during the height of the pandemic, such as patients asking if they could still walk around while on a ventilator.^18^ That said, this finding also reflects the paradox commonly observed in end-of-life care: most people indicate they would rather have a comfortable death, yet when they are offered invasive treatments to extend their life, they accept them.^19^ This is a limitation of ADs themselves, as they might not cover every possible outcome and could have been completed years before, making it unclear whether they are still an accurate record of the individual’s preferences. Even when a patient’s wishes are documented, they may not be followed, particularly when the instructions differ from their physician’s clinical opinion.^20^

While the country frame (and to a lesser extent, the COVID-19 prime) had some influence on what people chose to prioritize for their end-of-life care, it did not affect their self-reported likelihood to document their preferences in the near future. In both the UK and US conditions, slightly more than one-third of participants indicated they were likely or very likely to do so. This is a positive finding, as one concern about the framing of the ADRT is that it could deter people from making advance care plans at all. However, self-reported likelihood might not necessarily reflect whether people actually go on to write an AD, and only 3% of participants clicked on the link for more information, which is in line with statistics on UK completion rates.^2^

In the exploratory analysis of the additional variables collected, several had a significant influence on the choice between comfort or life-prolonging care. Increases in the age of the participants was linked to an increase in choosing comfort care, which is consistent with previous research on the treatment choices of cancer patients.^21^ Participants who were extremely worried about contracting COVID-19 and becoming seriously ill or dying of it were also more likely to choose comfort care, an effect that was driven by older participants. This measure had a small correlation with self-reported health quality, with those rating their health more poorly indicating they were more worried about COVID-19, which suggests these fears could reflect perceived vulnerability to the virus. However, the health measure itself did not affect choice of care, so it is unclear what exactly is driving this effect. Participants who indicated religion was very important to them were more likely to select comfort care, which might reflect certain belief systems.^22^ Again, when age groups were separated, this effect was significant only for the younger cohort. While it did not have a significant effect overall, gender did influence the preferences of younger participants, with men more likely to choose life-prolonging care. This is consistent with some research that has found men are more likely to receive treatment in the ICU in the last week of life,^23^ although it is unclear why this finding would not also be true for older participants. Other variables, such as ethnicity, education, or having documented end-of-life preferences, did not have an observable impact on patients’ choices. Interestingly, having private health insurance also did not influence participants’ choices, which suggests that the more aggressive treatments often seen in the private sector may be due to perverse incentives for the clinicians, rather than the preferences of the patients.^24^ However, it is important to note that these analyses were likely to be underpowered due to smaller subgroups of participants, and a lack of correction for multiple hypotheses testing could lead to false positives.

It is important to note that, although the different framing did influence participants’ choices, the country frame, COVID-19 prime, and the interaction between the two account for only 5% of the variance in their preferences for end-of-life care. This rises to just 9% when factors such as age, gender, and ethnicity are included, which suggests that there are more explanatory variables to be identified in future work.

This study has also 2 important limitations that may limit its generalizability. The first is the sample itself, which was not fully representative of the UK population and may therefore limit the generalizability of the findings. The participants were more likely to be younger, female, nonreligious, and more educated compared with the general population. In particular, age had a significant impact on participants’ choices, but with only 5.6% of the total sample aged 60 y or older, it was not possible to observe the effects of the experimental conditions on this subgroup in isolation. Moreover, the overwhelming preference for comfort-oriented care is surprising given most participants (71.2%) were younger than 40 y. Previous work has demonstrated that younger people are more likely to choose life-prolonging care,^25^ which suggests the sample studied here may have been unusual in some way. One factor could be education, with more than half of the sample having at least some university education, compared with one-third of the general population in England and Wales.^26^ Participants were also not screened for health conditions, and several studies have shown that the preferences of people with serious or terminal illnesses can differ from healthy populations.^27^ However, participants were asked to rate their health from excellent to poor (which has been demonstrated to be a reliable measure of health status)^28^ and whether they had ever been admitted to the ICU. Neither of these measures influenced participants’ choices between comfort or prolong. This study must be replicated with a more representative population, particularly focusing on a larger sample of older participants to understand how they were differentially influenced by the AD framing.

The second limitation is the design of the form that participants filled out. The order in which the questions were presented was not randomized, which could have influenced the way participants answered them. If participants had reflected on specific illnesses or medical treatments before stating their overall goal of care, it might have changed their preferences. Furthermore, to keep the 2 forms as similar as possible, the final design of the UK-style ADRT was quite different from the typical form template. This is because the form is designed only for those who wish to refuse treatment; there is no option for life-prolonging care. If participants were given the choice to simply fill in the form or not, which would be more reflective of real-life decision making, there could have been a very different outcome. However, this would have made the results from the 2 conditions less useful to compare. Clearly, completing an AD is not the same as choosing comfort care, so it is important to separate these 2 constructs in future studies. The participants were also never explicitly asked if they understood refusal of care to mean they would receive no treatment at all, and instead their choice of life-prolonging care is used as a proxy for this. This needs to be examined in more detail.

Even with these limitations, these findings have important implications for public policy with regard to documenting preferences for end-of-life care. The percentage of UK residents with an AD is far lower than many comparable Western countries, and it is likely that this disparity is driven in part by the way the question is frequently asked. Instead of comfort care being framed as an equal choice for treatment, it is instead framed as refusing treatment altogether. Just as patients tend to prefer taking action to doing nothing,^10^ it seems this framing discourages people from engaging with advance care planning. This is problematic, as there are many benefits to completing an AD, such as receiving care consistent with one’s preferences.^1^ The results from the US frame also suggest that people have a higher preference for comfort care than would be revealed in the UK system, which should be addressed to ensure people can receive the care they really want. This research also demonstrates that which questions are asked is just as important as how the questions are asked, as questions about specific illnesses received very different responses to questions about specific treatments. If this finding is robust to future replications, the UK ADRT should be reviewed and the framing of the questions reconsidered.

## Conclusions

This study is the first of its kind to experimentally examine the effect of framing comfort care as a refusal of treatment on people’s choices between comfort or life-prolonging care. While the majority of participants still chose comfort care and personal preferences played a role, this framing made people significantly more likely to choose life-prolonging care. This could have important policy implications, as it may be a factor explaining the unusually low rates of ADRT completion in the United Kingdom. This effect was exacerbated by priming participants to think about COVID-19, which suggests that living through a pandemic could paradoxically make people less likely to engage with advance care planning. If this is the case, it is crucial to find ways to better engage people in this area, particularly during health crises. While the effect of the UK frame also influenced choices for specific illnesses, the effect disappeared and preferences reversed when it came to specific treatments; participants in all conditions were more likely to accept aggressive treatments than to refuse. However, there are some important limitations of the study, particularly that the sample was not representative of the UK population in pertinent ways, such as education, religion, and especially age. Future work must be conducted with a more representative sample to explore how patients make decisions about their treatment and what influences the decisions of clinicians about their patients’ care.

## Supplemental Material

sj-docx-1-mdm-10.1177_0272989X231171139 – Supplemental material for End-of-Life Preferences: A Randomized Trial of Framing Comfort Care as Refusal of Treatment in the Context of COVID-19 Click here for additional data file. Supplemental material, sj-docx-1-mdm-10.1177_0272989X231171139 for End-of-Life Preferences: A Randomized Trial of Framing Comfort Care as Refusal of Treatment in the Context of COVID-19 by Juliet S. Hodges, Lilia V. Stoyanova and Matteo M. Galizzi in Medical Decision Making
