# Supplementary material for: End-of-Life Preferences: A Randomized Trial of Framing Comfort Care as Refusal of Treatment in the Context of COVID-19
Source: Med Decis Making. 2023 May 18;43(6):631–41. doi: 10.1177/0272989X231171139 (PMC10196681; doi:10.1177/0272989X231171139)
Supplement: sj-docx-1-mdm-10.1177_0272989X231171139 – Supplemental material for End-of-Life Preferences: A Randomized Trial of Framing Comfort Care as Refusal of Treatment in the Context of COVID-19 [file sj-docx-1-mdm-10.1177_0272989X231171139.docx]

**Supplementary material – full experiment instructions**

**Page 1 – overview and consent**

Thank you for choosing to participate in this study about Advance Decisions, also known as Living Wills.

The purpose of an Advance Decision is to give individuals the ability to make decisions about their medical treatment at the end of their life. These decisions are recorded in case, for health reasons, they become unable to make or communicate their wishes and are unlikely to regain the ability to do so.

As part of this research, we will ask you to complete a brief version of an Advance Decision. This includes questions about the overall goals of your care, and in case of specific conditions and treatments. Once you complete it, we will ask you some additional questions, including information about your experiences and views on health issues.

Though the form is based on a real Advance Decision, it is not real and the preferences you record here will not be added to your medical record.

All of your answers will be completely anonymous. If you feel uncomfortable, you can quit at any time and your data will be destroyed.

If you have any questions about the study, you can contact the researcher at [email address].

Do you consent to participate in the study?

**Page 2 – COVID-19 prime [2 versions – prime or no prime]**

*No COVID-19 prime*

Many people of varying ages die suddenly and unexpectedly, often alone and without leaving instructions for their end-of-life care.

The following questions will give you an opportunity to consider your preferences and specify your wishes for end-of-life care.

*COVID-19 prime*

The world is currently in the midst of the COVID-19 pandemic, caused by a novel coronavirus, SARS-CoV-2. To date, there have been almost 30 million cases and over 920,000 deaths worldwide.

This disease is characterised by a fever, cough and shortness of breath. The majority of people who contract the disease have mild to moderate cases, a category which includes all symptoms up to mild pneumonia. Severe cases include extensive lung damage and a lack of oxygen in the body, while in critical cases, patients can suffer with respiratory failure or multi-organ system dysfunction.

Patients with severe or critical cases of COVID-19 may be hospitalised, admitted to intensive care units and may even require mechanical ventilation to help them breathe. Estimates vary, but it has been suggested that around 2% of patients with the disease have died of it.

As a result, many people of varying ages have died suddenly and unexpectedly, often alone and without leaving instructions for their end-of-life care.

The following questions will give you an opportunity to consider your preferences and specify your wishes for end-of-life care.

**Page 3 – instructions [2 versions – US or UK]**

*US/AD version*

The form you are completing is an Advance Decision. You will be able to record your wishes for your care in general and for specific conditions and treatments, to take effect when you are no longer able to understand or communicate your preferences.

You can choose for your healthcare providers to focus on maximising your comfort, but it may shorten the length of your life.

Or you can choose for your healthcare providers to focus on helping you live as long as possible, but it may increase your pain and suffering.

**Please complete the following as if you were completing a real Advance Decision that would become part of your medical record.**

*UK/ADRT version*

The form you are completing is an Advance Decision to Refuse Treatment. You will be able to record your wishes for your care in general and for specific conditions and treatments, to take effect when you are no longer able to understand or communicate your preferences.

If you refuse treatment, your healthcare providers will focus on maximising your comfort, but it may shorten the length of your life.

If you accept treatment, your healthcare providers will focus on helping you live as long as possible, but it may increase your pain and suffering.

**The standard for care in the UK is to make every effort to prolong a patient's life, unless they have documented their wish to refuse treatment.**

**Please complete the following as if you were completing a real Advance Decision that would become part of your medical record.**

**Page 4 – instructions continued [2 versions – US or UK]**

*US/AD version*

Advance Decision:

The following healthcare treatment instructions are intended to provide clear and convincing evidence of my wishes to be followed when I lack the capacity to understand, make, or communicate my treatment decisions.

This will take effect when and only when I lack the ability to understand, make, or communicate a choice regarding a health or personal care decision, as verified by the doctor in charge of my care.

*UK/ADRT version*

Advance Decision to Refuse Treatment:

The following healthcare treatment instructions are intended to provide clear and convincing evidence of my wishes to be followed when I lack the capacity to understand, make, or communicate my treatment decisions.

This will take effect when and only when I lack the ability to understand, make, or communicate a choice regarding a health or personal care decision, as verified by the doctor in charge of my care.

**Page 5 – overall goal of care question [2 versions and answer order counterbalanced]**

If I have a condition where I have no reasonable expectation of recovery or chance of regaining a meaningful quality of life, my instructions for the overall goal of my care are as follows:

*US/AD version*

- I want my health care providers and agent to pursue treatments that help relieve my pain and suffering, even if that means that I might not live as long.
- I want my health care providers and agent to pursue treatments that help me to live as long as possible, even if that means I might have more pain or suffering.

*UK/ADRT version*

- I would like to exercise my right to refuse treatment. I want my healthcare providers and agent to only pursue treatments that help relieve my pain and suffering, even if that means that I might not live as long.
- I do not want to refuse treatment and I would like to accept the care available to me. I want my healthcare providers and agent to pursue treatments that help me to live as long as possible, even if that means I might have more pain or suffering.

**Page 6 – specific illness: dementia question [2 versions and answer order counterbalanced]**

If I have one of the following conditions, and I have no reasonable expectation of recovery or chance of regaining a meaningful quality of life, my instructions are as follows:

If I have been diagnosed with any type of dementia

*US/AD version*

- I want my health care providers and agent to pursue treatments that help relieve my pain and suffering, even if that means that I might not live as long.
- I want my health care providers and agent to pursue treatments that help me to live as long as possible, even if that means I might have more pain or suffering.

*UK/ADRT version*

- I would like to exercise my right to refuse treatment. I want my healthcare providers and agent to only pursue treatments that help relieve my pain and suffering, even if that means that I might not live as long.
- I do not want to refuse treatment and I would like to accept the care available to me. I want my healthcare providers and agent to pursue treatments that help me to live as long as possible, even if that means I might have more pain or suffering.

**Page 7 – specific illness: brain injury question [2 versions and answer order counterbalanced]**

If I sustain a brain injury (including, but not limited to, stroke, vegetative and minimally conscious states)

*US/AD version*

- I want my health care providers and agent to pursue treatments that help relieve my pain and suffering, even if that means that I might not live as long.
- I want my health care providers and agent to pursue treatments that help me to live as long as possible, even if that means I might have more pain or suffering.

*UK/ADRT version*

- I would like to exercise my right to refuse treatment. I want my healthcare providers and agent to only pursue treatments that help relieve my pain and suffering, even if that means that I might not live as long.
- I do not want to refuse treatment and I would like to accept the care available to me. I want my healthcare providers and agent to pursue treatments that help me to live as long as possible, even if that means I might have more pain or suffering.

**Page 8 – specific illness: CNS disease question [2 versions and answer order counterbalanced]**

If I am diagnosed with a disease of the central nervous system (including, but not limited to, motor neurone disease, Parkinson's disease and Huntingdon's disease)

*US/AD version*

- I want my health care providers and agent to pursue treatments that help relieve my pain and suffering, even if that means that I might not live as long.
- I want my health care providers and agent to pursue treatments that help me to live as long as possible, even if that means I might have more pain or suffering.

*UK/ADRT version*

- I would like to exercise my right to refuse treatment. I want my healthcare providers and agent to only pursue treatments that help relieve my pain and suffering, even if that means that I might not live as long.
- I do not want to refuse treatment and I would like to accept the care available to me. I want my healthcare providers and agent to pursue treatments that help me to live as long as possible, even if that means I might have more pain or suffering.

**Page 9 – specific illness: terminal illness question [2 versions and answer order counterbalanced]**

If I am diagnosed with a terminal illness (including, but not limited to, advanced cancer, lung disease or heart disease)

*US/AD version*

- I want my health care providers and agent to pursue treatments that help relieve my pain and suffering, even if that means that I might not live as long.
- I want my health care providers and agent to pursue treatments that help me to live as long as possible, even if that means I might have more pain or suffering.

*UK/ADRT version*

- I would like to exercise my right to refuse treatment. I want my healthcare providers and agent to only pursue treatments that help relieve my pain and suffering, even if that means that I might not live as long.
- I do not want to refuse treatment and I would like to accept the care available to me. I want my healthcare providers and agent to pursue treatments that help me to live as long as possible, even if that means I might have more pain or suffering.

**Page 10 – specific treatment: CPR question [2 versions and answer order counterbalanced]**

If I have a condition where I have no reasonable expectation of recovery or chance of regaining a meaningful quality of life, my instructions for these specific treatments are as follows:

Cardiopulmonary resuscitation (CPR; manual chest compressions performed to restore blood circulation and breathing)

*US/AD version*

- I do not want cardiopulmonary resuscitation (CPR) to be performed if my heart stops beating, even if performing CPR might prolong my life.
- I request cardiopulmonary resuscitation (CPR) to prolong my life if my heart stops beating, even if CPR might increase my pain or suffering.

*UK/ADRT version*

- I would like to exercise my right to refuse treatment. I do not want cardiopulmonary resuscitation (CPR) to be performed if my heart stops beating, even if performing CPR might prolong my life.
- I do not want to refuse treatment and I would like to accept the care available to me. I request cardiopulmonary resuscitation (CPR) to prolong my life if my heart stops beating, even if CPR might increase my pain or suffering.

**Page 11 – specific treatment: ICU question [2 versions and answer order counterbalanced]**

Intensive care unit (ICU) admission (hospital unit that provides specialised equipment, services, and monitoring for critically ill patients, such as higher staffing-to-patient ratios and ventilator support)

*US/AD version*

- I do not want to be admitted to the intensive care unit (ICU), even if it might prolong my life.
- I want to be admitted to the intensive care unit (ICU) to prolong my life, even if it might increase my pain or suffering.

*UK/ADRT version*

- I would like to exercise my right to refuse treatment. I do not want to be admitted to the intensive care unit (ICU), even if it might prolong my life.
- I do not want to refuse treatment and I would like to accept the care available to me. I want to be admitted to the intensive care unit (ICU) to prolong my life, even if it might increase my pain or suffering.

**Page 12 – specific treatment: mechanical ventilator question [2 versions and answer order counterbalanced]**

Mechanical ventilator use (a general term to describe machines that assist spontaneous breathing, often using either a mask or a breathing tube)

*US/AD version*

- I do not want a mechanical ventilator to be used, even if it might prolong my life.
- I request the use of a mechanical ventilator to prolong my life, even if it might increase my pain or suffering.

*UK/ADRT version*

- I would like to exercise my right to refuse treatment. I do not want a mechanical ventilator to be used, even if it might prolong my life.
- I do not want to refuse treatment and I would like to accept the care available to me. I request the use of a mechanical ventilator to prolong my life, even if it might increase my pain or suffering.

**Page 13 – specific treatment: dialysis question [2 versions and answer order counterbalanced]**

Dialysis (kidney filtration by machine)

*US/AD version*

- I do not want dialysis to be performed on me, even if it might prolong my life.
- I request dialysis to prolong my life, even if it might increase my pain or suffering.

*UK/ADRT version*

- I would like to exercise my right to refuse treatment. I do not want dialysis to be performed on me, even if it might prolong my life.
- I do not want to refuse treatment and I would like to accept the care available to me. I request dialysis to prolong my life, even if it might increase my pain or suffering.

**Page 14 – specific treatment: feeding tube question [2 versions and answer order counterbalanced]**

Feeding tube insertion (devices used to provide nutrition to patients who cannot swallow, inserted either through the nose and oesophagus into the stomach or directly into the stomach through the belly)

*US/AD version*

- I do not want to have a feeding tube inserted, even if it might prolong my life.
- I request feeding tube insertion to prolong my life, even if it might increase my pain or suffering.

*UK/ADRT version*

- I would like to exercise my right to refuse treatment. I do not want to have a feeding tube inserted, even if it might prolong my life.
- I do not want to refuse treatment and I would like to accept the care available to me. I request feeding tube insertion to prolong my life, even if it might increase my pain or suffering.

**Page 15 – health attitudes: documentation of end-of-life wishes**

Thank you for completing the Advance Decision.

Have you documented your preferences for end-of-life care in any way? Please indicate if you have any of the following (select all that apply).

- An advance decision or living will
- A lasting power of attorney
- An organ donor or registration card
- Other (please specify)
- I have not documented my end-of-life preferences

**Page 16 – likelihood to document of end-of-life wishes**

How likely are you to document your preferences for end-of-life care in the near future?

- Very likely
- Likely
- Neither likely nor unlikely
- Unlikely
- Very unlikely

**Page 17 – behavioural measure of clicking through to NHS website**

If you would like more information or links to templates to create a real Advance Decision, [click here](https://www.nhs.uk/conditions/end-of-life-care/advance-decision-to-refuse-treatment/) to visit the NHS page on Advance Decisions (link will open in a new tab).

Now please continue on to the final questions about you, your experiences and views on health issues.

**Page 18 – age [data also collected via participants’ accounts on Prolific Academic]**

How old are you?

- 18-29
- 30-39
- 40-49
- 50-59
- 60-69
- 70+

**Page 19 – gender [data also collected via participants’ accounts on Prolific Academic]**

What is your gender?

- Female
- Male
- Non-binary
- Other

**Page 20 - ethnicity**

What is your ethnic group?

- Asian / Asian British
- Black / African / Caribbean / Black British
- Mixed / multiple ethnic groups
- White
- Other ethnic group

**Page 21 – education**

What is your highest level of education?

- Secondary education (e.g. GCSEs)
- A levels
- Undergraduate degree (e.g. BSc, BA)
- Postgraduate degree (e.g. MA, MSc)
- Doctoral degree (e.g. PhD)
- Vocational qualification(s) (e.g. BTEC, NVQ)
- Professional qualifications (e.g. for teaching, nursing, accountancy)
- Other
- No formal qualifications

**Page 22 - religion**

What is your religious affiliation?

- Buddhist
- Christian
- Hindu
- Jewish
- Muslim
- Sikh
- Other
- None

**Page 23 – religious importance**

How important is religion in your life?

- Very important
- Somewhat important
- Not important at all

**Page 24 – understanding of standard end-of-life care**

In a situation where a patient cannot communicate their wishes and has not given instructions for their end-of-life care, what do you think healthcare providers would prioritise when making decisions about the patient's care?

- Maximising comfort
- Prolonging life

**Page 25 – attitudes to healthcare providers**

Which statement best describes your attitude towards medical care?

- The patient should take complete control
- The patient should have more control than the doctor
- The patient and the doctor should share control
- The doctor should have more control than the patient
- The doctor should take complete control

**Page 26 – private health insurance**

Do you have private health insurance?

- Yes, I pay for my own policy (or covered by someone else's personal policy, e.g. spouse)
- Yes, through my employer (or covered by someone else's policy from their employer, e.g. spouse)
- No

**Page 27 – health measure**

In general, would you say your health is:

- Excellent
- Very good
- Good
- Fair
- Poor
- I prefer not to say

**Page 28 – experience of an ICU**

Have you ever been a patient in an intensive care unit?

- Yes
- No
- I prefer not to say

**Page 29 – experience of death of a loved one**

Have you ever experienced the death of a loved one?

- Yes
- No
- I prefer not to say

**Page 30 – COVID-19 concerns**

How worried are you that you will contract Covid-19 (coronavirus)?

- Extremely worried
- Moderately worried
- Slightly worried
- Not at all worried
- I prefer not to say

**Page 31 – COVID-19 concerns**

How worried are you that you will become seriously ill or die from Covid-19 (coronavirus)?

- Extremely worried
- Moderately worried
- Slightly worried
- Not at all worried
- I prefer not to say

**Page 32 – COVID-19 concerns**

Have you had, or do you suspect you have had, Covid-19 (coronavirus)?

- Yes
- No
- I prefer not to say

[If yes] How severe was your case of Covid-19 (coronavirus)?

- Asymptomatic
- Mild
- Moderate
- Severe (e.g. hospitalised)
- Critical (e.g. hospitalised, required support with breathing, organ failure)
- I prefer not to say

**Page 33 – COVID-19 concerns**

How worried are you about friends or family becoming seriously ill or dying from Covid-19 (coronavirus)?

- Extremely worried
- Moderately worried
- Slightly worried
- Not at all worried
- I prefer not to say

**Page 34 – COVID-19 concerns**

Has anyone you know had, or suspects they have had, coronavirus (Covid-19)?

- Yes, one person
- Yes, more than one person
- No
- I prefer not to say

[If yes, one person] How severe was their case of Covid-19 (coronavirus)?

- Asymptomatic
- Mild
- Moderate
- Severe (e.g. hospitalised)
- Critical (e.g. hospitalised, required support with breathing, organ failure)
- Died of the disease or related complications
- I prefer not to say

[If yes, more than one person] Of the people you know who have had Covid-19 (coronavirus), how severe was the most extreme case?

- Asymptomatic
- Mild
- Moderate
- Severe (e.g. hospitalised)
- Critical (e.g. hospitalised, required support with breathing, organ failure)
- Died of the disease or related complications
- I prefer not to say
